# Opioid and Naloxone Prescribing Following Insertion of Prompts in the Electronic Health Record to Encourage Compliance With California State Opioid Law

**DOI:** 10.1001/jamanetworkopen.2022.9723

**Published:** 2022-05-02

**Authors:** Lewei Duan, Ming-Sum Lee, John L. Adams, Adam L. Sharp, Jason N. Doctor

**Affiliations:** 1 Department of Research and Evaluation, Kaiser Permanente Southern California, Pasadena; 2 Department of Cardiology, Kaiser Permanente Los Angeles Medical Center, Los Angeles, California; 3 Kaiser Permanente Center for Effectiveness and Safety Research, Pasadena, California; 4 Department of Health System Science, Kaiser Permanente Bernard J. Tyson School of Medicine, Pasadena, California; 5 Department of Emergency Medicine, Kaiser Permanente Los Angeles Medical Center, Los Angeles, California; 6 Price School of Public Policy, University of Southern California, Los Angeles

## Abstract

**Importance:**

Opioid addiction or dependency is a serious crisis in the US that affects public health as well as social and economic welfare. The State of California passed Assembly Bill (AB) 2760 in 2018 that mandates the coprescription of naloxone and opioids for patients with a high overdose risk.

**Objective:**

To assess whether the AB 2760–based electronic prompts were associated with increased naloxone orders for opioid users and reduced opioid prescribing when integrated into the practitioner workflow.

**Design, Setting, and Participants:**

This cohort study used interrupted time series mixed models to evaluate data obtained from the regional integrated health care system Kaiser Permanente Southern California (KPSC) from January 1, 2018, to December 31, 2019. Clinician participants were continuously employed at KPSC during the study period and ordered an opioid analgesic for eligible patients in 2018. Patient participants were KPSC members aged 18 years or older who received an opioid analgesic prescription during the study period. A series of AB 2760–based electronic prompts were integrated into the KPSC electronic health record system on December 27, 2018. The prompts are triggered or activated when 1 or more opioid prescribing conditions, defined in the AB 2760, are met at outpatient visits. Data were analyzed from January 8, 2021, to September 15, 2021.

**Exposures:**

Assembly Bill 2760–based electronic prompts for outpatient opioid prescriptions in the electronic health record system.

**Main Outcomes and Measures:**

Primary outcomes were changes in outpatient naloxone order rates among patients who were prescribed opioids and changes in outpatient opioid prescribing rates. Secondary outcomes were total morphine milligram equivalents (MMEs) ordered per prescriber-month, prompts-targeted objectives, and unintended consequences. Risk for opioid abuse among 3 types of patients was also assessed.

**Results:**

The 6515 eligible clinicians (mean [SD] age, 45.9 [9.43] years; 3604 men [55.3%]) included in the study served 500 711 unique patients in 1 903 289 outpatient encounters (mean [SD] age, 60.4 [15.67] years; 1 121 004 women [58.9%]) in which an opioid analgesic was prescribed. Naloxone order rate increased from 2.0% in December 2018 to 13.2% in January 2019 and then continued to increase to 27.1% in December 2019. Outpatient opioid prescribing rates decreased by 15.1% (rate ratio [RR], 0.85; 95% CI, 0.83-0.87) per prescriber-month when the electronic prompts were implemented. The postimplementation trend increased by 0.7% per prescriber-month (RR, 1.01; 95% CI, 1.01-1.01); the overall trend was still decreasing. The total MMEs per prescriber-month decreased by 7.8% (RR, 0.92; 95% CI, 0.89-0.96) after implementation of the prompts. The postimplementation trend tapered off. Other safe opioid prescribing measures also improved after implementation (decreases in concomitant muscle relaxants orders [RR, 0.94; 95% CI, 0.89-1.00], initial [RR, 0.86; 0.83-0.89] and renewal [RR, 0.65; 95% CI, 0.62-0.69] opioid orders, and long-term high-dose orders [RR, 0.96; 95% CI, 0.94-0.98]).

**Conclusions and Relevance:**

This study found an association between implementation of AB 2760–based prompts and increased naloxone order rate; improved opioid prescribing measures (ie, decreased concomitant muscle relaxants orders, initial and renewal opioid orders, and long-term high-dose orders), except monthly median MMEs; and reduced opioid prescribing. The findings suggest that opioid overdose risks can be mitigated by encouraging safe prescribing habits.

## Key Points

**Question:**

Does the extra barrier of using clinical decision support to accommodate state policy reduce opioid prescribing by clinicians?

**Findings:**

In this cohort study of 6515 clinicians serving 500 711 unique patients, the opioid prescribing rate decreased and naloxone order rate increased; all opioid prescribing measures also improved (ie, decreased concomitant muscle relaxants orders, initial and renewal opioid orders, and long-term high-dose orders) after the launch of electronic prompts.

**Meaning:**

Findings of this study suggest that adding decision support prompts to the practitioner workflow that encourage safe prescribing habits can mitigate opioid overdose risks.

## Teaser

This cohort study examines changes in naloxone and opioid prescribing rates in the outpatient setting at 1 integrated health care system after the launch of decision-support prompts in the electronic health record (HER) in compliance with the State of California Assembly Bill 2760.

## Introduction

The US is in the middle of an opioid crisis.^1^ Most people with opioid addiction or dependency were first exposed through a pain management prescription.^2^ Despite ongoing efforts to curb the crisis, fatal overdoses continue to increase.^3^ Government officials, health care practitioners, and investigators have been searching for methods to better regulate opioid prescriptions.^4,5^ The State of California passed Assembly Bill (AB) 2760, effective January 1, 2019, to address the opioid crisis. This law mandates prescribers to offer naloxone, an opioid antagonist,^6,7^ or another comparable drug approved by the US Food and Drug Administration, with the opioid prescription^8^ to patients who are at high risk for opioid overdose (eMethods in the Supplement). The law further requires prescribers to educate these patients and their caregivers about overdose prevention and the use of naloxone. By encouraging greater knowledge and accessibility of naloxone among patients at high risk of overdose, AB 2760 serves as an overdose prevention strategy.

Over the past 3 decades, decision support prompts in electronic health record (EHR) systems have been used to facilitate clinical practice.^9^ A recent study found an association between EHR alerts and changes in postoperative opioid prescriptions,^10^ but to nudge clinician behavior toward achieving legislative goals, a broader study incorporating a wide range of clinical settings is warranted. At Kaiser Permanente Southern California (KPSC), 16 categories of prompts were implemented to support AB 2760–based clinical decision-making for outpatient opioid prescription. When a prescription order is entered into the EHR, an electronic prompt is triggered when 1 or more conditions, which are defined in AB 2760, are met. The prompts produce safety alerts that explain the risks of opioid prescribing, remind clinicians to order naloxone, and list recommended actions that clinicians can choose to follow or override.

The primary objective of this study was to evaluate whether the AB 2760–based prompts were associated with increased naloxone orders for opioid users and reduced opioid prescribing when integrated into the practitioner workflow. By comparing prescribing practices before and after implementation of the prompts, we could evaluate whether naloxone orders would increase or, conversely, whether outpatient opioid orders would decrease. We hypothesized that clinicians would adopt the opioid prescribing behaviors that are targeted by the prompts and that changing prescribing patterns would be associated with lower risk of opioid overuse; that is, clinicians would exercise greater caution when treating initial opioid users and long-term high-dose users as well as when dealing with patients’ renewal orders.

## Methods

This cohort study obtained retrospective EHR data from KPSC, a regional integrated health care system consisting of 15 medical centers and 235 medical offices in Southern California. More than 7600 physicians and 26 000 nurses serve 4.6 million members of KPSC, an ethnically and socioeconomically diverse population that is broadly representative of Southern California.^11^ A customized EHR system (Epic Systems) was implemented across all KPSC-covered service areas. The study protocol was approved by the KPSC Institutional Review Board, which waived the informed consent requirement because of the observational nature of the study. We followed the Strengthening the Reporting of Observational Studies in Epidemiology (STROBE) reporting guideline.

### Exposure

The prompts for outpatient opioid prescriptions were integrated into the EHR on December 27, 2018, and are activated or triggered by 1 or more of the following conditions: (1) the prescriber writes an opioid order that will result in the patient receiving a total opioid morphine milligram equivalents (MMEs) of 90 or greater per day, (2) the patient has concurrent active prescriptions of an opioid and benzodiazepines, and (3) the patient has had an opioid overdose diagnosis within the previous 2 years. These prompts automatically exclude patients with terminal illness, those who were discharged to a hospice, and those who had declined naloxone at baseline. We also excluded patients with cancer at baseline. The prompts could be overridden, allowing clinicians to retain their autonomy in practice.

### Study Sample

Using the National Drug Code numbers for opioids that were issued by the Centers for Disease Control and Prevention,^12^ we searched the EHR system for KPSC members aged 18 years or older who received an opioid analgesic prescription between January 1, 2018, and December 31, 2019. The index date was the date of active opioid prescription, and baseline was 1 year before the prescription date.

All clinicians (nonresidents) who were included in this study had a valid federal Drug Enforcement Administration number and placed outpatient opioid prescription orders for the identified patients in 2018. Each eligible clinician was employed at KPSC continuously throughout 2018 and 2019, to ensure equal exposure to the prompts.

Race and ethnicity data were self-identified by patients and clinicians. Participants who did not think they fit in any of the race and ethnicity categories (American Indian or Alaska Native, Asian, Hispanic, Native Hawaiian or Other Pacific Islander, Non-Hispanic Black, and Non-Hispanic White) were identified under the other or unknown category.

### Outcomes

The primary outcomes were changes in outpatient naloxone order rates among patients who received an opioid prescription and in outpatient opioid prescribing rates after the implementation of the prompts. The secondary outcomes were (1) prescribed dose levels, defined as total MMEs ordered per prescriber-month and monthly median MMEs ordered per prescriber per patient encounter; (2) prompts-targeted objectives, defined as opioid prescriptions for patients who were at risk for opioid overdose and/or had concomitant prescription of benzodiazepines; and (3) unintended consequences, defined as concomitant prescription of muscle relaxants with opioid prescriptions. Furthermore, we checked whether the prompts changed opioid prescribing rates for 3 types of patients who were at risk for opioid abuse: those with initial opioid orders, renewal opioid orders, or long-term high-dose orders (eMethods in the Supplement). Unintended consequences and risk for opioid abuse were considered as other safe opioid prescribing measures because they were not the target criterion for the prompts.

### Statistical Analysis

The descriptive statistics for patient and clinician characteristics included in the study were frequencies and percentages for categorical variables and means and SDs for continuous variables. We used bar charts to illustrate the firing patterns of the prompts as well as the monthly frequency and order rates of naloxone prescription. We used an interrupted time series^13^ study design with segmented regression and generalized linear mixed models (eMethods in the Supplement), including a clinician random-effect intercept, to estimate trends and explore the association of the prompts with opioid prescribing patterns of clinicians. Models were adjusted for clinician clustering, nested within medical center areas. We used zero-inflated negative binomial distribution for overdispersed count outcome variables that contained excessive zeros, and we used the Tweedie distribution for continuous outcomes that had nonignorable positive mass at zero.^14^ Model results were reported on the rate ratio (RR) scale.

Models were further adjusted for the following variables: clinician age, sex, race and ethnicity, and type of medical degree as well as whether the clinicians were adult primary care physicians (eMethods in the Supplement). We also adjusted for the number of years of employment at KPSC, which served as a proxy for the clinicians’ familiarity with the organization’s EHR system. Models tested whether the AB 2760–based prompts were associated with an immediate change in opioid prescription volume at the time of the interruption and with any long-term change in the clinician prescribing patterns.

We assessed the sensitivity of the findings through changes in the mean monthly prescription rate per clinician in the year after vs before the implementation of the prompts using a pre-post design with a physician random-effect intercept. To address the heterogeneity of treatment effect, we incorporated an interaction term to evaluate whether clinicians with unique characteristics would have responded to the prompts differently. These characteristics included age, sex, and medical degree. We also conducted a subgroup analysis involving physicians only, to assess whether responses to the prompts varied between primary care physicians and non–primary care physicians. We used linear contrasts to estimate the different implications of the prompts for clinicians with different characteristics.

A 2-sided *P* < .05 was considered to be statistically significant. All statistical analyses were conducted in SAS, version 9.4 (SAS Institute Inc) and R, version 4.0.4 (R Foundation for Statistical Computing^15^). Data were analyzed from January 8, 2021, to September 15, 2021.

## Results

A total of 6515 eligible clinicians were included in the study, with a mean (SD) age of 45.9 (9.43) years at the beginning of the study and a mean (SD) duration of employment at KPSC of 11.4 (9.09) years (Table 1). Of these clinicians, 2911 were women (44.7%) and 3604 were men (55.3%); most individuals were of Asian race and ethnicity (n = 2803 [43.0%]), earned an MD (doctor of medicine) or DO (doctor of osteopathic medicine) degree (5683 [87.2%]), and practiced as primary care physicians (2850 [43.7%]). Between 2018 and 2019, these clinicians served 500 711 unique patients in 1 903 289 outpatient encounters in which an opioid analgesic was prescribed.

**Table 1.** Clinician and Patient Characteristics

Characteristic	Descriptive statistics, No. (%)
Clinician characteristics	
No. of clinicians	6515
Age at the beginning of the study, mean (SD), y	45.9 (9.43)
Years of employment at KPSC, mean (SD)	11.4 (9.09)
Sex	
Female	2911 (44.7)
Male	3604 (55.3)
Race and ethnicity^a^	
American Indian or Alaska Native	184 (2.8)
Asian	2803 (43.0)
Hispanic	600 (9.2)
Non-Hispanic Black	250 (3.8)
Non-Hispanic White	2583 (39.6)
Other or unknown^b^	95 (1.5)
Clinician type	
Physician (MD or DO)	5683 (87.2)
Midlevel practitioner (PA or NP)	630 (9.7)
Other specialist (DPM, CNM, or DDS)	202 (3.1)
Primary care physician	
Yes	2850 (43.7)
No	3665 (56.3)
Patient characteristics	
No. of patients	500 711
Patient encounters	1 903 289
Age at encounter, mean (SD), y	60.4 (15.67)
Sex	
Female	1 121 004 (58.9)
Male	782 277 (41.1)
Other^c^	8 (0.002)
Race and ethnicity^a^	
American Indian or Alaska Native	6254 (0.3)
Asian	73 817 (3.9)
Hispanic	537 061 (28.2)
Native Hawaiian or Other Pacific Islander	7282 (0.4)
Non-Hispanic Black	225 920 (11.9)
Non-Hispanic White	1 028 457 (54.0)
Other or unknown^b^	24 498 (1.3)
Overdose history	
Yes	404 121 (21.2)
No	1 499 168 (78.8)

Abbreviations: CNM, certified nurse midwife; DDS, doctor of dental surgery; DO, doctor of osteopathic medicine; DPM, doctor of podiatric medicine; MD, doctor of medicine; NP, nurse practitioner; PA, physician assistant.

^a^ Race and ethnicity were self-identified.

^b^ Other or unknown category was used when the individuals did not think they fit any of the race and ethnicity categories presented.

^c^ Other sex included transgender and those who did not identify as female or male.

Patients who received opioid prescriptions had a mean (SD) age of 60.4 (15.67) years and consisted of 1 121 004 women (58.9%), 782 277 men (41.1%), and 8 (0.002%) who were categorized under other sex (eg, transgender, those who did not identify as female or male). Most patients had a non-Hispanic White race and ethnicity (1 028 457 [54.0%]). Among all eligible encounters resulting in an opioid prescription, 404 121 (21.2%) involved patients with a history of overdose within 2 years before the encounter.

### The Prompts

Between December 27, 2018, and December 31, 2019, the prompts were activated 812 571 times. Of these, a total of 263 484 prompts (32.4%) were triggered for orders placed by the identified clinicians, and 132 111 prompts (16.3%) were triggered for visits from eligible patients (eFigure 1 in the Supplement). The frequency of activated prompts reached its peak in January 2019, before gradually decreasing and leveling off.

### Outpatient Naloxone Orders and Order Rates

**Figure 1.**
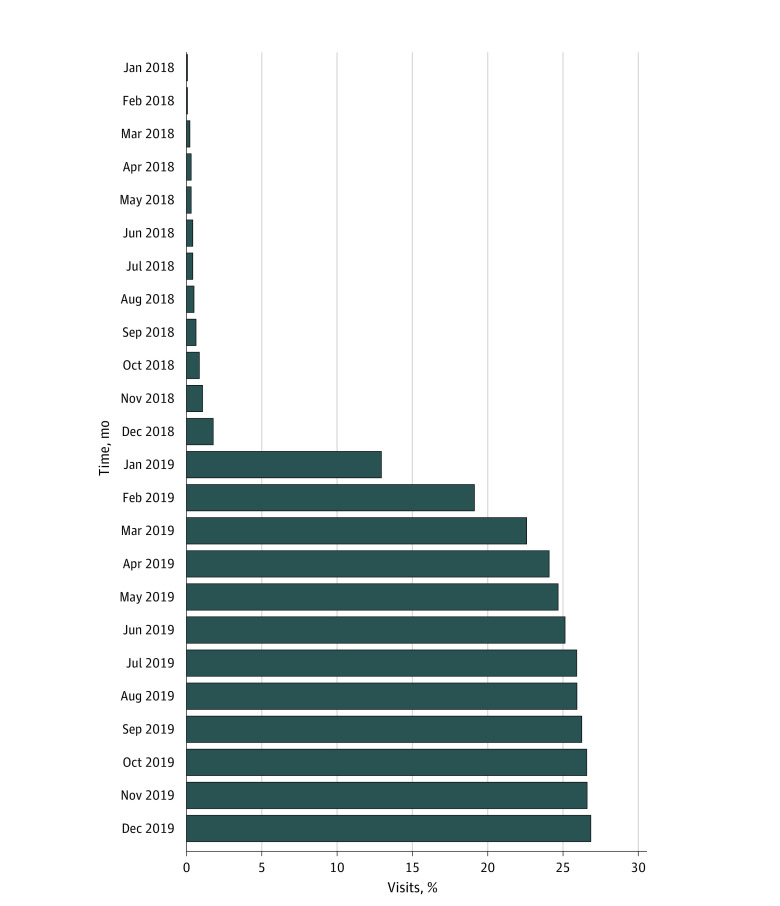
Baseline Naloxone Order Rate at the Encounter With Opioid Prescription

The pattern of outpatient naloxone orders was consistent with the pattern of the prompts when they were triggered (eFigure 2 in the Supplement). Naloxone order rates on a month-by-month basis throughout 2018 and 2019 sharply increased after implementation of the prompts (Figure 1). Although a rate of less than 1% was observed for each month before September 2018, this rate doubled to 2.0% in December 2018, increased to 13.2% in January 2019, and continued to increase to 27.1% in December 2019, with a steadily climbing trajectory. Patients may have had access to naloxone through other means.

### Outpatient Opioid Prescription Rates

**Figure 2.**
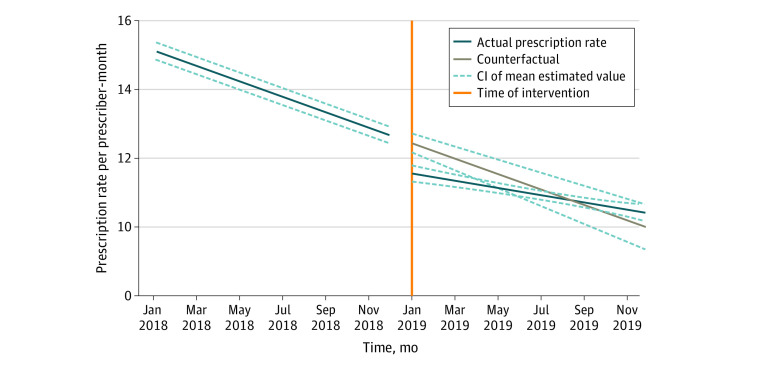
Interrupted Time Series Graph for Opioid Prescribing Rate per Prescriber-Month Time series data were graphed using mixed-effect model estimates. The scale did not go to 100%.

All outcome measures experienced moderate decreasing trends in 2018, and clinicians prescribed fewer opioids in 2019 (Table 2, Figure 2; eTable 1 in the Supplement). Opioid orders per prescriber-month decreased by 15.1% (RR, 0.85; 95% CI, 0.83-0.87). Before implementation of the prompts, opioid orders had been decreasing by 1.6% per prescriber-month (RR, 0.98; 95% CI, 0.98-0.99). The postimplementation trend increased by 0.7% per prescriber-month (RR, 1.01; 95% CI, 1.01-1.01). Tapering trends remained after implementation, but the declining trends before implementation suggested that prescribers had been vigilant in practicing safe opioid prescription.

**Table 2.** Mixed Models With Interrupted Time Series Analysis of Opioid-Prescribing Measures Before and After Implementation of Prompts

	RR (95% CI)^a^		
	Immediate change^b^	Preimplementation change^b^	Postimplementation change^b^
Quantity and dose of opioid prescription			
Opioid prescription	0.85 (0.83-0.87)^c^	0.98 (0.98-0.99)^c^	1.01 (1.01-1.01)^c^
Total MME	0.92 (0.89-0.96)^c^	0.98 (0.98-0.98)^c^	1.00 (1.00-1.00)
Median MME per order	1.11 (1.06-1.17)^c^	0.99 (0.99-0.99)	0.99 (0.99-1.00)^d^
Prompts-targeted objectives			
Overdose history	0.88 (0.85-0.91)^c^	0.98 (0.98-0.99)^c^	1.00 (1.00-1.00)
Concomitant benzodiazepines	0.79 (0.76-0.83)^c^	0.97 (0.97-0.97)^c^	1.00 (0.99-1.00)
Unintended consequence			
Concomitant muscle relaxants	0.94 (0.89-1.00)^d^	0.99 (0.99-0.99)^c^	0.99 (0.99-0.99)^c^
Patient type at risk for opioid abuse			
Initial opioid order user	0.86 (0.83-0.89)^c^	0.98 (0.98-0.98)^c^	1.01 (1.01-1.01)^c^
Renewal opioid order user	0.65 (0.62-0.69)^c^	0.95 (0.95-0.95)^c^	1.03 (1.03-1.04)^c^
Long-term high-dose order user	0.96 (0.94-0.98)^c^	0.99 (0.99-0.99)^c^	1.00 (0.99-1.00)^c^

Abbreviations: MME, morphine milligram equivalent; RR, rate ratio.

^a^ Model estimates are reported as a scale of RR.

^b^ Models were adjusted for within-clinician clustering, nested within medical center areas. Models were also adjusted for clinician age, sex, race and ethnicity, and type of medical degree as well as whether clinicians were primary care physicians. Clinician-level data were collected monthly.

^c^ *P* < .001.

^d^ *P* < .05.

Total MMEs per prescriber-month decreased by 7.8% (RR, 0.92; 95% CI, 0.89-0.96) after implementation of the prompts, which was comparable to an immediate reduction of 377.8 MMEs per prescriber-month. The postimplementation trend remained on a decreasing trajectory (eFigure 3 in the Supplement). Median MMEs per order increased by 11.2% (RR, 1.11; 95% CI, 1.06-1.17) immediately, although the postimplementation trend leveled off slightly (RR, 0.99; 95% CI, 0.99-1.00). A possible explanation may be that fewer orders for low-dose prescriptions were made after the implementation. Prescribers seemed to have responded to the prompts by curtailing low-dose opioid prescriptions for patients whose pain was manageable via alternative means and by keeping high-dose opioid orders for patients who were in greater need.

Implementation of the prompts was associated with an immediate reduction in overdose history (RR, 0.88; 95% CI, 0.85-0.91) as well as concomitant prescription of benzodiazepines and opioids (RR, 0.79; 95% CI, 0.76-0.83). Although not an objective of the prompts, an immediate reduction in the prescription of opioids with concomitant muscle relaxants was found (RR, 0.94; 95% CI, 0.89-1.00), suggesting an association between implementation of the prompts and enhanced, safer prescribing practices.

Immediately after the implementation, opioid orders that were placed for initial opioid users per prescriber-month decreased (RR, 0.86; 95% CI, 0.83-0.89), clinicians exercised greater caution when renewing an opioid order (RR, 0.65; 95% CI, 0.62-0.69), and long-term high-dose orders reduced moderately (RR, 0.96; 95% CI, 0.94-0.98). We observed small changes in postimplementation trends and tapering trends overall. A pre-post analysis with monthly data indicated that the EHR prompts were associated with safer opioid prescribing practice (eTable 2 in the Supplement).

### Subgroup Analysis

Compared with 2018, the 2019 opioid orders per prescriber-year decreased by 23.2% (RR, 0.77; 95% CI, 0.76-0.78) (eTable 3 in the Supplement). All other outcome measures for safe opioid prescribing practices (concomitant muscle relaxants order, initial and renewal opioid order, and long-term high-dose order) showed substantial improvement after the implementation of the prompts. Physicians reacted to the prompts according to their unique characteristics. After the implementation, female physicians experienced a decline in their prescribing rates of 24.8% (RR, 0.76; 95% CI, 0.75-0.78), but no significant changes in opioid prescribing rates were observed among male physicians. All outcome measures improved markedly for physicians (ie, decreased concomitant muscle relaxants orders, initial and renewal opioid orders, and long-term high-dose orders), but not for midlevel practitioners and other specialists. All age groups showed improved opioid prescribing practices, but physicians aged 51 to 65 years showed less improvement (RR, 0.96; 95% CI, 0.92-1.00) (eTable 4 in the Supplement). Among physicians, primary care physicians responded to the implementation differently from non–primary care physicians (RR, 0.75 [95% CI, 0.73-0.76] vs 1.06 [95% CI, 1.03-1.09]; *P* < .001), except in the dose per order (RR, 0.94 [95% CI, 0.91-0.97] vs 0.93 [95% CI, 0.89-0.97]; *P* < .001) (eTable 5 in the Supplement).

## Discussion

This study evaluated changes in naloxone order and opioid prescribing patterns after the implementation of electronic prompts at KPSC to improve compliance with AB 2760. We found that naloxone order rates substantially increased and opioid prescribing rates decreased immediately after the implementation. Several insights from behavioral economics and social psychology may help explain these findings.

First, clinicians may change their prescribing behaviors out of regard for an injunctive norm; that is, they evaluate their own response to the prompts based on a socially established standard for appropriate prescribing.^4^ Second, AB 2760 and its prompts create psychological friction, meaning that prescribers may avoid ordering opioids because of the sludge effect,^16^ defined as the number of procedural obstacles to overcome before being able to prescribe an opioid. Third, we found overall safer opioid prescribing behaviors (eg, reduced concomitant muscle relaxant orders, initial and renewal opioid orders, and long-term high-dose orders) regardless of whether the patients met the criteria targeted by the prompts, suggesting that being observed (Hawthorne effect^17^) played a role in changing prescribing patterns. Physicians knew their prescribing was monitored. The organizational scrutiny of prescriber responses to the prompts was likely a factor in prescriber compliance with the reminders, thereby facilitating safer prescribing practices.

We observed decreasing preimplementation trends in opioid prescribing rates and safer opioid prescription practices, which suggested that clinicians were aware of the harm of overprescribing (anticipatory effect). The tapering trend before implementation may have left little room for further reduction (floor effect). The pattern of the changes in prescribing behavior was consistent with the pattern of the triggering of the prompts: strong initial changes, and then a return to baseline levels. These results suggest that prescribing practices can be prompted or nudged, but further interventions will be necessary for sustained adjustments. To avoid alert fatigue, future work needs to strengthen guideline-concordant actions that inhibit triggering an alert. Some alerts, such as justification alerts, can engender reputational concerns when poor prescribing justifications are given.^18^ Clinicians may be motivated to avoid such alerts, which can prevent alert fatigue.

We found differences in prescribing patterns by clinician characteristics. First, opioid prescribing rates decreased considerably among female clinicians, but not for male clinicians, after implementation of the prompts. Male clinicians demonstrated less change in use of decision support tools for prescribing. Second, physicians adopted safer opioid prescribing behavior after the prompts launched, but there was no similar change for midlevel practitioners or other specialists. One explanation for this finding is that most midlevel practitioners in the study may be required to serve patients with a greater need of an opioid prescription, but further research is needed. Third, after the implementation of the prompts, opioid prescribing behavior changed across age groups, but those aged 51 to 65 years had only modest changes in prescribing behavior. Given that this cohort of clinicians likely started medical practice between 1996 and 2011, during the aggressive marketing of oxycontin, they were likely more open to opioid prescribing. Fourth, primary care physicians were more susceptible to the recommendation from the prompts compared with non–primary care physicians. Further work is needed to address the plausible margins for the reduction of opioid orders on the basis of physician specialty.

The design of the EHR system at KPSC seemed to have minimized variations in staffing. We adjusted the models for variation at the clinician level, and the results should be interpreted as indicative of how prescribing patterns changed overall for a given clinician. We found that the organization-level standardization of EHR across clinics was associated with minimal variation among facilities. Thus, the specific processes in the prompts that KPSC integrated into the practitioner workflow merit further reflection.

We also found an association between the AB 2760–based decision support prompts and increased naloxone orders. The prompts at KPSC allow prescribers to exercise discretion, and overriding the prompts does not signify noncompliance nor imply good or bad practice. Alert fatigue or lack of clinical relevance may underlie some overriding,^19^ but overly fast tapering of opioids is nevertheless poor practice.^20^ Encouraging safer prescribing practices might involve offering behavioral interventions that have been associated with successful antibiotic prescribing.^21^

Additional research is needed to end the opioid crisis. A health care pipeline needs to be developed to encourage alternative pain management methods, such as counseling or using nonopioid medication. Studies are also needed to change prescribing practices for patients with inadequate health care access and those with uninsured or underinsured status.

### Limitations

This study has several limitations. First, it used retrospective data (no randomization). Second, there was no control group because the profile of opioid use in California made it difficult to find a suitable comparator. Third, some of the variability in clinician prescribing behaviors may be explained by patient characteristics and patient-clinician interactions. Fourth, clinicians who left KPSC during the study period were excluded from the analysis, although intent-to-treat analysis can be used to partially address potential selection bias. Fifth, changes in prescribing rate may be attributable to fewer patients reporting pain at visits and not necessarily fewer orders issued to patients with pain. In addition, patients who received a naloxone prescription may never fill it. Moreover, the unmeasured confounders may threaten the validity of the treatment effect estimation. Sixth, the findings may not be generalizable to non-KPSC prescribers.

## Conclusions

This cohort study found that opioid prescribing prompts, as clinical decision support tools, that were embedded in the EHR system at KPSC were associated with enhanced clinician compliance with state law (AB 2760 in California). Naloxone order rate increased after implementation of the prompts, and all opioid prescribing measures improved (decreased concomitant muscle relaxants orders, initial and renewal opioid orders, and long-term high-dose orders), except for the median MMEs per prescriber-month. The findings suggest that it is feasible to mitigate opioid overdose risks by encouraging safe prescribing habits using behavioral insights. Nudges, when incorporated into the EHR, can change clinician behaviors. A combination of behavioral interventions and strategic designs should be applied in the health care delivery system to facilitate the implementation of public health policies and to sustain the outcomes. Ultimately, the goal of curbing opioid abuse is to prevent the problem upstream.

## Notes

Supplement. **eMethods.** Supplemental Description of Methods **eFigure 1.** Frequency of AB2760-based BAPs Triggered During Study Period **eFigure 2.** Frequency of Naloxone Prescription During Study Period **eFigure 3.** Interrupted Time Series Graph for Total MMEs Ordered per Prescriber Based on Mixed Effect Models **eTable 1.** Full Report of Mixed Model With Interrupted Time Series Analysis of Opioid Prescribing Measures Before and After Implementation of AB2670-based BPAs **eTable 2.** Sensitivity Analysis: Pre-Post Analysis With Monthly Data **eTable 3.** Pre-Post Analysis (Annually) of Changes in Clinicians’ Prescription Behavior **eTable 4.** Pre-Post Analysis (Annually) of Changes in Clinicians’ Prescription Behavior by Age Groups **eTable 5.** Subgroup Analysis (Physicians Only): Pre-Post Analysis (Annually) of Changes in Clinicians’ Prescription Behavior
