# Supplementary material for: Opioid and Naloxone Prescribing Following Insertion of Prompts in the Electronic Health Record to Encourage Compliance With California State Opioid Law
Source: JAMA Netw Open. 2022 May 2;5(5):e229723. doi: 10.1001/jamanetworkopen.2022.9723 (PMC9062689; doi:10.1001/jamanetworkopen.2022.9723)
Supplement: Supplement. — eMethods. Supplemental Description of Methods eFigure 1. Frequency of AB2760-based BAPs Triggered During Study Period eFigure 2. Frequency of Naloxone Prescription During Study Period eFigure 3. Interrupted Time Series Graph for Total MMEs Ordered per Prescriber Based on Mixed Effect Models eTable 1. Full Report of Mixed Model With Interrupted Time Series Analysis of Opioid Prescribing Measures Before and After Implementation of AB2670-based BPAs eTable 2. Sensitivity Analysis: Pre-Post Analysis With Monthly Data eTable 3. Pre-Post Analysis (Annually) of Changes in Clinicians’ Prescription Behavior eTable 4. Pre-Post Analysis (Annually) of Changes in Clinicians’ Prescription Behavior by Age Groups eTable 5. Subgroup Analysis (Physicians Only): Pre-Post Analysis (Annually) of Changes in Clinicians’ Prescription Behavior [file jamanetwopen-e229723-s001.pdf]

# Supplementary Online Content

Duan L, Lee MS, Adams JL, Sharp AL, Doctor JN. Opioid and naloxone prescribing following insertion of prompts in the electronic health record to encourage compliance with California state opioid law. *JAMA Netw Open*. 2022;5(5):e229723. doi:10.1001/jamanetworkopen.2022.9723

**eMethods.** Supplemental Description of Methods

**eFigure 1.** Frequency of AB2760-based BAPs Triggered During Study Period

**eFigure 2.** Frequency of Naloxone Prescription During Study Period

**eFigure 3.** Interrupted Time Series Graph for Total MMEs Ordered per Prescriber Based on Mixed Effect Models

**eTable 1.** Full Report of Mixed Model With Interrupted Time Series Analysis of Opioid Prescribing Measures Before and After Implementation of AB2670-based BPAs

**eTable 2.** Sensitivity Analysis: Pre-Post Analysis With Monthly Data

**eTable 3.** Pre-Post Analysis (Annually) of Changes in Clinicians' Prescription Behavior

**eTable 4.** Pre-Post Analysis (Annually) of Changes in Clinicians' Prescription Behavior by Age Groups

**eTable 5.** Subgroup Analysis (Physicians Only): Pre-Post Analysis (Annually) of Changes in Clinicians' Prescription Behavior

This supplementary material has been provided by the authors to give readers additional information about their work.

© 2022 Duan L et al. *JAMA Network Open.*

## **eMethods.** Supplemental Description of Methods

### A. Definition of high-risk opioid overdose

The law requires that the prescribers offer a prescription for naloxone hydrochloride among patients who receive a dosage is at least 90 milligram morphine equivalents, an opioid medication prescribed concurrently with a prescription for benzodiazepine, and who presents with an increased risk of overdose as determined by the patient's history. Throughout this paper we refer to patients that meet any or all of these criteria "high risk".

### B. Definition of Endpoints

For the primary endpoints, we define naloxone prescription rate as orders placed per month; naloxone possession rate as percentage of patients who were prescribed with naloxone at discharge within the previous year. We define outpatient opioid prescription rate as the number of opioid orders for patients at discharge per prescriber-month or per prescriber-year in different model settings. We assessed the immediate and long-term effects of the prompts on clinicians' prescription patterns measured monthly. We also assessed average annual changes, comparing pre- and post-intervention at clinicians' level.

For the secondary endpoints, we classify a concomitant prescription as when an opioid and a specified medication were placed either 30 days before or after the order of the other. For other endpoints, we define initial opioid orders as when patients who were prescribed an opioid without having previously had such a prescription at least 90 days prior to the index date; we define renewal orders as when those patients who received an opioid prescription within 90 days of the index date, but had not been prescribed opioids more than 90 days prior to the index date; we define chronically high-dose orders when dosage levels reached MME $\geq$ 50: this group had also received two or more prescriptions with two different start dates within the 90 days preceding the index date, as well as at least one prescription between 91 and 180 days prior to the index date.

### C. Statistical Model

We used an interrupted time series study design with segmented regression and generalized linear mixed models.

$$f\big(E(Y_{it})\big) = \beta_0 + \delta_1 \mathrm{I_t} + \delta_2 \mathrm{T_t} + \delta_3 \mathrm{I_t} * \mathrm{T_t} + \hat{X}_{it}\hat{\beta}_{it} + \mu_i + \epsilon_{it},$$

$where\ \epsilon_{\mathrm{it}} \sim \mathrm{N}(0, \sigma_\varepsilon^2)$ and $\mu_{\mathrm{it}} \sim \mathrm{N}\big(0, \sigma_\mu^2\big)$;

i: Prescriber; t: time (month);

The analysis takes the form of an interrupted time series mixed model with segmented regression using monthly repeated measures. The outcome is a function of the expected value of $Y_{it}$, where i is the prescriber, and t is time. The right side of the equation is composed of the following elements: 1) the intercept term; 2) an indicator variable for when the prompt warning was put in place; 3) a linear term of a trend that occurs over the course of the data period; 4) the change of the slope term at the point of the intervention. The $X$ and β vectors are all the fixed covariates at prescriber level that might be additional explanatory factors for the outcome variable; 5) a random

© 2022 Duan L et al. *JAMA Network Open.*

intercept term for each prescriber; and 6) an error term that gives us the error in every time period within each prescriber.

As a sensitivity analysis, we analyzed the model with a quadratic time term. The non-linear model yielded compatible estimates as the linear model.

**D. Definition of Variables**

We define an adult primary care provider as one who specializes in internal medicine, family medicine, or general practice. Types of degree fall into the following categories: Physician (Medical Doctor, Osteopathic Doctor), mid-level providers (Physician Assistant or Nurse Practitioner), and other specialists (Podiatrist, Certified nurse midwife, and Doctor of Dental Surgery).

© 2022 Duan L et al. *JAMA Network Open.*

**eFigure 1.** Frequency of AB2760-based BAPs Triggered During Study Period

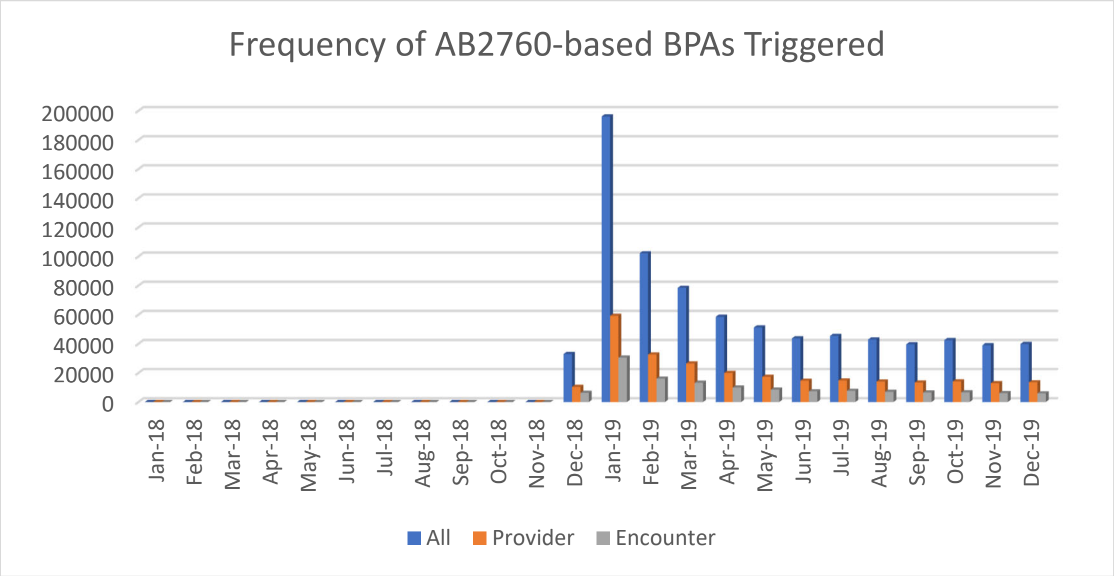


The AB2760-based BPAs is launched on December 17, 2019.

© 2022 Duan L et al. *JAMA Network Open.*

**eFigure 2.** Frequency of Naloxone Prescription During Study Period

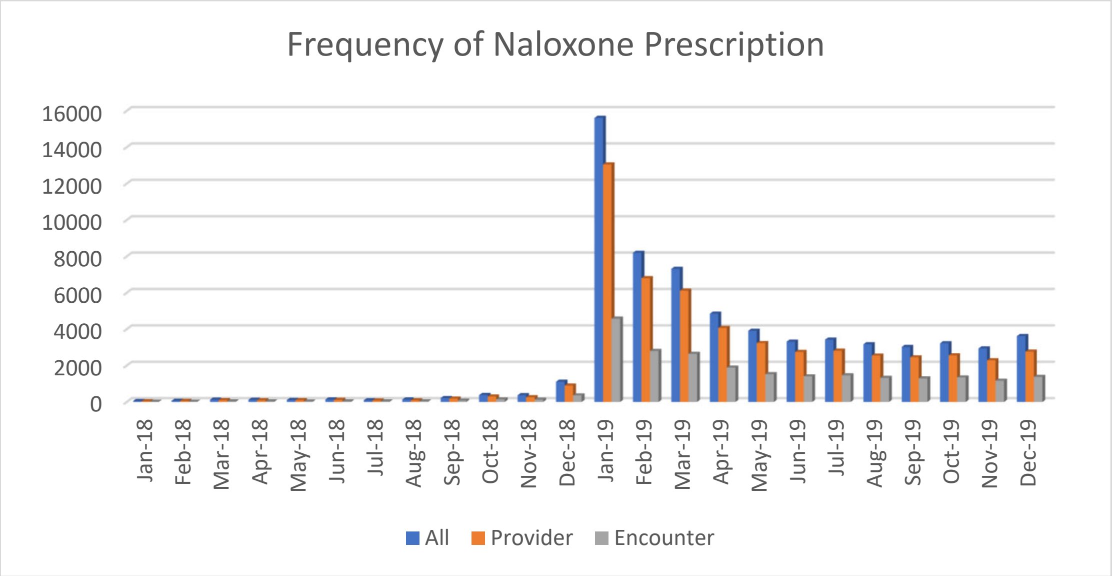


The frequency of naloxone prescription is defined as the count of encounter led to naloxone prescription.

© 2022 Duan L et al. *JAMA Network Open.*

**eFigure 3.** Interrupted Time Series Graph for Total MMEs Ordered per Prescriber Based on Mixed Effect Models

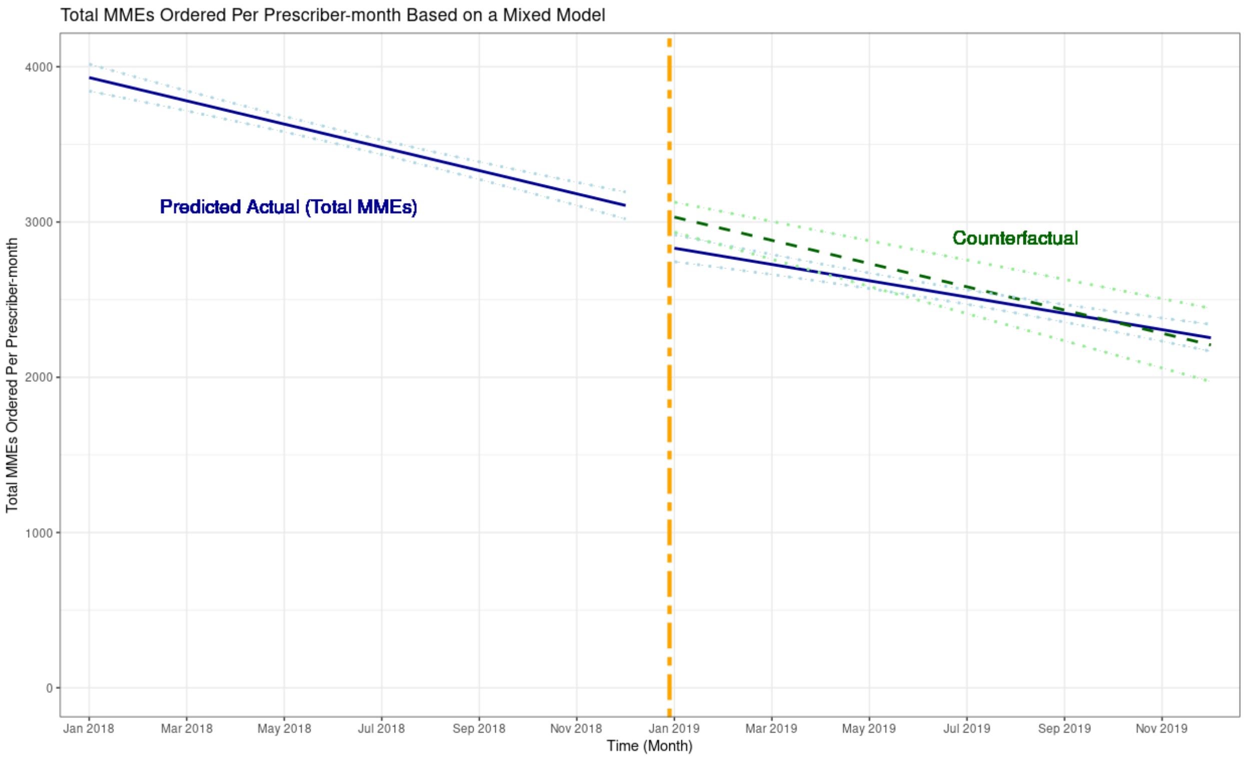


Time series were graphed based on model estimates. Blue solid line represents predicted opioid prescription rate; green dashed line represents counterfactual; light blue and light green dotted line represent confidence intervals around the mean prediction values; orange two-dashed vertical line represents time of intervention.

**eTable 1.** Full Report of Mixed Model With Interrupted Time Series Analysis of Opioid Prescribing Measures Before and After Implementation of AB2670-based BPAs

| | Quantity and Dosage of Opioid Prescription | | | The Prompt Targeted Objective | |
|---|---|---|---|---|---|
| Outcome | Opioid Prescription | Total MME | Median MME per order | Overdose history | Concomitant Benzodiazepines |
| Covariates | | | | | |
| Immediate Effect (prompts) | 0.849 (0.831 to 0.867) | 0.922 (0.89 to 0.955) | 1.112 (1.059 to 1.168) | 0.881 (0.853 to 0.91) | 0.791 (0.757 to 0.826) |
| Time trend (Month, Pre) | 0.984 (0.983 to 0.985) | 0.979 (0.977 to 0.981) | 0.991 (0.989 to 0.994) | 0.983 (0.982 to 0.985) | 0.968 (0.966 to 0.969) |
| Change in trend (Month, Post) | 1.007 (1.005 to 1.008) | 1.001 (0.998 to 1.003) | 0.994 (0.991 to 0.997) | 1 (0.998 to 1.003) | 0.997 (0.994 to 1) |
| Age (Year) | 0.994 (0.987 to 1.001) | 0.998 (0.989 to 1.007) | 1.003 (0.997 to 1.008) | 0.997 (0.989 to 1.004) | 0.996 (0.988 to 1.004) |
| Years employed in KPSC | 0.991 (0.983 to 0.998) | 0.986 (0.977 to 0.995) | 0.994 (0.988 to 0.999) | 0.986 (0.979 to 0.994) | 0.992 (0.984 to 1) |
| Gender (Male) | 1.364 (1.255 to 1.481) | 1.188 (1.074 to 1.314) | 0.884 (0.831 to 0.94) | 1.593 (1.467 to 1.729) | 1.421 (1.302 to 1.55) |
| Race (Asian) | 0.975 (0.737 to 1.289) | 0.84 (0.598 to 1.18) | 0.894 (0.726 to 1.1) | 0.882 (0.668 to 1.165) | 0.923 (0.686 to 1.242) |
| Race (Hispanic) | 1.206 (0.895 to 1.624) | 1.118 (0.778 to 1.607) | 0.999 (0.801 to 1.247) | 0.94 (0.698 to 1.265) | 1.01 (0.737 to 1.386) |
| Race (Non-Hispanic Black) | 1.33 (0.944 to 1.874) | 1.065 (0.702 to 1.618) | 0.876 (0.679 to 1.131) | 1.089 (0.774 to 1.532) | 1.158 (0.806 to 1.663) |
| Race (Non-Hispanic White) | 1.04 (0.79 to 1.369) | 0.827 (0.592 to 1.156) | 0.843 (0.688 to 1.034) | 0.861 (0.655 to 1.132) | 0.904 (0.676 to 1.211) |
| Race (Other or Unknown) | 0.779 (0.508 to 1.194) | 0.595 (0.354 to 1.002) | 0.778 (0.566 to 1.069) | 0.739 (0.483 to 1.132) | 0.771 (0.491 to 1.211) |
| Medical Degree (DPM/CNM) | 0.481 (0.367 to 0.629) | 0.429 (0.309 to 0.596) | 0.812 (0.665 to 0.993) | 0.323 (0.245 to 0.426) | 0.236 (0.174 to 0.32) |
| Medical Degree (MD/OD) | 0.642 (0.546 to 0.755) | 0.524 (0.43 to 0.638) | 0.673 (0.597 to 0.76) | 0.663 (0.564 to 0.779) | 0.737 (0.621 to 0.875) |
| Specialty (Specialised) | 0.315 (0.289 to 0.344) | 0.174 (0.157 to 0.194) | 0.494 (0.463 to 0.527) | 0.226 (0.207 to 0.247) | 0.234 (0.213 to 0.256) |

© 2022 Duan L et al. *JAMA Network Open.*

**eTable 1. Continued**

| | Unintended Consequence | Risk of Opioid Abuse | | |
|---|---|---|---|---|
| Outcome | Concomitant Muscle Relaxants | Initial Opioid Order | Renewal Opioid Order | Chronically High Dose Order |
| Covariates | | | | |
| Immediate Effect (prompts) | 0.941 (0.891 to 0.995) | 0.857 (0.83 to 0.885) | 0.652 (0.615 to 0.691) | 0.958 (0.935 to 0.981) |
| Time trend (Month, Pre) | 0.987 (0.985 to 0.99) | 0.981 (0.98 to 0.983) | 0.951 (0.948 to 0.953) | 0.989 (0.987 to 0.99) |
| Change in trend (Month, Post) | 0.989 (0.985 to 0.992) | 1.01 (1.007 to 1.012) | 1.031 (1.027 to 1.036) | 0.995 (0.993 to 0.997) |
| Age (Year) | 1 (0.992 to 1.008) | 0.995 (0.988 to 1.003) | 0.985 (0.979 to 0.992) | 1.001 (0.991 to 1.011) |
| Years employed in KPSC | 0.981 (0.972 to 0.989) | 0.987 (0.98 to 0.995) | 0.992 (0.985 to 0.998) | 0.989 (0.978 to 0.999) |
| Gender (Male) | 1.486 (1.359 to 1.626) | 1.254 (1.153 to 1.363) | 1.48 (1.379 to 1.588) | 1.663 (1.483 to 1.866) |
| Race (Asian) | 0.91 (0.673 to 1.23) | 1.253 (0.943 to 1.663) | 0.858 (0.676 to 1.088) | 0.893 (0.604 to 1.32) |
| Race (Hispanic) | 1.087 (0.789 to 1.499) | 1.799 (1.33 to 2.435) | 0.934 (0.725 to 1.204) | 0.936 (0.618 to 1.42) |
| Race (Non-Hispanic Black) | 1.373 (0.949 to 1.986) | 1.864 (1.317 to 2.638) | 1.186 (0.886 to 1.587) | 1.048 (0.65 to 1.689) |
| Race (Non-Hispanic White) | 0.876 (0.652 to 1.179) | 1.509 (1.141 to 1.994) | 0.954 (0.755 to 1.205) | 0.759 (0.517 to 1.115) |
| Race (Other or Unknown) | 0.679 (0.426 to 1.082) | 1.128 (0.731 to 1.739) | 0.727 (0.505 to 1.048) | 0.649 (0.357 to 1.178) |
| Medical Degree (DPM/CNM) | 0.11 (0.079 to 0.152) | 0.461 (0.351 to 0.607) | 0.742 (0.59 to 0.933) | 0.164 (0.11 to 0.246) |
| Medical Degree (MD/OD) | 0.403 (0.339 to 0.48) | 0.639 (0.543 to 0.753) | 0.633 (0.551 to 0.727) | 0.593 (0.472 to 0.744) |
| Specialty (Specialised) | 0.185 (0.168 to 0.203) | 0.702 (0.643 to 0.767) | 0.81 (0.752 to 0.873) | 0.069 (0.062 to 0.078) |

The model was adjusted for within-provider clustering, nested within medical center areas. Data is collected at clinician's level.

Model estimates are reported as a scale of rate ratio (RR).

Column names represent outcomes measured; raw names represent covariates in the model.

© 2022 Duan L et al. *JAMA Network Open.*

**eTable 2.** Sensitivity Analysis: Pre-Post Analysis With Monthly Data

| | | **Rate Ratio (95% CI)** [a] |
|---|---|---|
| | | **Pre vs Post** |
| **Quantity and Dosage of Opioid Prescription** | | |
| | Opioid Prescription | 0.793 (0.789 to 0.797)[b] |
| | Total MME | 0.723 (0.717 to 0.729)[b] |
| | Median MME per order | 0.862 (0.841 to 0.885)[b] |
| **The Prompt Targeted Objective** | | |
| | Overdose history | 0.73 (0.724 to 0.736)[b] |
| | Concomitant Benzodiazepines | 0.52 (0.514 to 0.526)[b] |
| **Unintended Consequence** | | |
| | Concomitant Muscle Relaxants | 0.667 (0.657 to 0.676)[b] |
| **Risk of Opioid Abuse** | | |
| | Initial Opioid Order | 0.819 (0.812 to 0.826)[b] |
| | Renewal Opioid Order | 0.64 (0.628 to 0.651)[b] |
| | Chronically High Dose Order | 0.757 (0.752 to 0.762)[b] |

[a] Data is collected monthly at clinicians' level.
[b] p<0.001.

© 2022 Duan L et al. *JAMA Network Open.*

**eTable 3.** Pre-Post Analysis (Annually) of Changes in Clinicians' Prescription Behavior

| | | All, RR (95% CI)[a] | Gender, RR (95% CI)[a] | | Medical Degree Types, RR (95% CI)[a] | | |
|---|---|---|---|---|---|---|---|
| | | | Female[b] | Male | Physician[b] | Mid-level Provider | Specialist |
| **Quantity and Dosage of Opioid Prescription** | | | | | | | |
| | Opioid Prescription | 0.768 (0.757 to 0.779)[c] | 0.762 (0.746 to 0.779)[c] | 1.012 (0.983 to 1.042)[e] | 0.767 (0.755 to 0.779)[c] | 0.961 (0.915 to 1.01)[e] | 1.188 (1.087 to 1.299)[c,e] |
| | Total MME | 0.724 (0.712 to 0.736)[c] | 0.743 (0.725 to 0.762)[c] | 0.954 (0.922 to 0.986)[e] | 0.722 (0.71 to 0.735)[c] | 0.97 (0.913 to 1.029)[e] | 1.319 (1.161 to 1.5)[c,e] |
| | Median MME per order | 0.862 (0.841 to 0.885)[c] | 0.922 (0.889 to 0.957)[c] | 0.881 (0.837 to 0.927)[c] | 0.859 (0.836 to 0.883)[c] | 0.953 (0.876 to 1.038) | 1.272 (1.107 to 1.461)[d,e] |
| **The Prompt Targeted Objective** | | | | | | | |
| | Overdose history | 0.705 (0.693 to 0.717)[c] | 0.693 (0.675 to 0.712)[c] | 1.029 (0.994 to 1.066)[e] | 0.706 (0.693 to 0.719)[c] | 0.971 (0.913 to 1.032)[e] | 1.066 (0.934 to 1.217)[e] |
| | Concomitant Benzodiazepines | 0.501 (0.49 to 0.511)[c] | 0.486 (0.47 to 0.501)[c] | 1.055 (1.011 to 1.1)[d,e] | 0.497 (0.486 to 0.508)[c] | 1.064 (0.986 to 1.147)[e] | 1.181 (0.992 to 1.405)[e] |
| **Unintended Consequence** | | | | | | | |
| | Concomitant Muscle Relaxants | 0.652 (0.639 to 0.666)[c] | 0.636 (0.616 to 0.656)[c] | 1.046 (1.003 to 1.091)[d,e] | 0.653 (0.639 to 0.667)[c] | 0.963 (0.896 to 1.035)[e] | 1.36 (1.095 to 1.688)[d,e] |
| **Risk of Opioid Abuse** | | | | | | | |
| | Initial Opioid Order | 0.791 (0.779 to 0.803)[c] | 0.777 (0.76 to 0.795)[c] | 1.031 (1 to 1.063)[d,e] | 0.787 (0.775 to 0.8)[c] | 0.988 (0.939 to 1.04)[e] | 1.257 (1.144 to 1.381)[c,e] |
| | Renewal Opioid Order | 0.602 (0.59 to 0.614)[c] | 0.591 (0.573 to 0.61)[c] | 1.031 (0.99 to 1.074)[e] | 0.596 (0.584 to 0.609)[c] | 1.034 (0.97 to 1.101)[e] | 1.205 (1.073 to 1.353)[d,e] |
| | Chronically High Dose Order | 0.682 (0.668 to 0.696)[c] | 0.69 (0.669 to 0.712)[c] | 0.978 (0.94 to 1.019)[e] | 0.692 (0.678 to 0.707)[c] | 0.848 (0.786 to 0.914)[c,e] | 0.819 (0.679 to 0.988)[d] |

[a] Data is collected annually at clinicians' level. Rate ratios (RR) represent the ratio of post-intervention rate / pre-intervention rate. Interaction term was used for the comparison between clinicians' characteristics.
[b] The reference group in the subgroup analysis.
[c] p<0.001 for rate ratio in annual pre-post analysis.
[d] p<0.05 for rate ratio in annual pre-post analysis.
[e] p<0.001 for difference between the group with specified trait and reference group. P value for difference is derived from multiple comparison method.

© 2022 Duan L et al. *JAMA Network Open.*

**eTable 4.** Pre-Post Analysis (Annually) of Changes in Clinicians' Prescription Behavior by Age Groups

| | Age (Years), Rate Ratio (95% CI)[a] | | | |
|---|---|---|---|---|
| | Age <=35 | Age 36-50 | Age 51-65 | Age >=66 |
| **Quantity and Dosage of Opioid Prescription** | | | | |
| Opioid Prescription | 0.818 (0.789 to 0.848)[b] | 0.788 (0.709 to 0.876)[b] | 0.959 (0.921 to 0.999)[c] | 0.865 (0.825 to 0.906)[b] |
| Total MME | 0.78 (0.747 to 0.815)[b] | 0.85 (0.74 to 0.976)[c] | 0.933 (0.889 to 0.979)[c] | 0.878 (0.832 to 0.928)[b] |
| Median total MME per order | 0.88 (0.824 to 0.94)[b] | 0.929 (0.781 to 1.104) | 0.973 (0.904 to 1.048) | 0.989 (0.91 to 1.075) |
| **The Prompt Targeted Objective** | | | | |
| Overdose history | 0.745 (0.713 to 0.778)[b] | 0.84 (0.729 to 0.968)[c] | 0.965 (0.919 to 1.014)[c] | 0.874 (0.826 to 0.925)[b] |
| Concomitant Benzodiazepines | 0.557 (0.528 to 0.588)[b] | 0.908 (0.765 to 1.077) | 0.895 (0.843 to 0.95)[b] | 0.845 (0.79 to 0.905)[b] |
| **Unintended Consequence** | | | | |
| Concomitant Muscle Relaxants | 0.71 (0.673 to 0.748)[b] | 0.812 (0.68 to 0.969)[c] | 0.93 (0.876 to 0.987)[c] | 0.85 (0.794 to 0.91)[b] |
| **Risk of Opioid Abuse** | | | | |
| Initial Opioid Order | 0.838 (0.807 to 0.871)[b] | 0.803 (0.716 to 0.901)[b] | 0.963 (0.923 to 1.006) | 0.875 (0.833 to 0.92)[b] |
| Renewal Opioid Order | 0.646 (0.615 to 0.678)[b] | 0.877 (0.74 to 1.04) | 0.943 (0.892 to 0.996)[c] | 0.867 (0.813 to 0.924)[b] |
| Chronically High Dose Order | 0.743 (0.706 to 0.782)[b] | 0.827 (0.7 to 0.977)[c] | 0.932 (0.88 to 0.987)[c] | 0.84 (0.787 to 0.897)[b] |

[a] Data is collected annually at clinicians' level. Age groups represent clinicians' age (in year) at the beginning of the study. Rate ratios represent the ratio of post-intervention rate / pre-intervention rate for the specific group. Interaction term was used for the comparison between clinicians' age groups.
[b] p<0.001.
[c] p<0.05.

© 2022 Duan L et al. *JAMA Network Open.*

**eTable 5.** Subgroup Analysis (Physicians Only): Pre-Post Analysis (Annually) of Changes in Clinicians' Prescription Behavior

| | All, RR (95% CI)[a] | Primary Care Physician, RR (95% CI)[a] | |
|---|---|---|---|
| | | PCP[b] | Non-PCP |
| **Quantity and Dosage of Opioid Prescription** | | | |
| Opioid Prescription | 0.768 (0.757 to 0.779)[c] | 0.747 (0.732 to 0.762)[c] | 1.063 (1.032 to 1.094)[c,d] |
| Total MME | 0.722 (0.71 to 0.734)[c] | 0.717 (0.702 to 0.732)[c] | 1.021 (0.985 to 1.059)[d] |
| Median MME per order | 0.861 (0.838 to 0.884)[c] | 0.938 (0.912 to 0.965)[c] | 0.93 (0.892 to 0.971)[c] |
| **The Prompt Targeted Objective** | | | |
| Overdose history | 0.706 (0.693 to 0.719)[c] | 0.696 (0.68 to 0.713)[c] | 1.036 (0.998 to 1.076)[d] |
| Concomitant Benzodiazepines | 0.497 (0.486 to 0.508)[c] | 0.481 (0.467 to 0.494)[c] | 1.091 (1.042 to 1.142)[c,d] |
| **Unintended Consequence** | | | |
| Concomitant Muscle Relaxants | 0.653 (0.639 to 0.667)[c] | 0.618 (0.602 to 0.634)[c] | 1.185 (1.133 to 1.241)[c,d] |
| **Risk of Opioid Abuse** | | | |
| Initial Opioid Order | 0.788 (0.777 to 0.801)[c] | 0.724 (0.71 to 0.739)[c] | 1.198 (1.163 to 1.234)[c,d] |
| Renewal Opioid Order | 0.597 (0.585 to 0.61)[c] | 0.543 (0.527 to 0.559)[c] | 1.217 (1.166 to 1.269)[c,d] |
| Chronically High Dose Order | 0.692 (0.678 to 0.707)[c] | 0.73 (0.712 to 0.749)[c] | 0.842 (0.805 to 0.881)[c,d] |

[a] Data is collected annually at clinicians' level. Rate ratios (RR) represent the ratio of post-intervention rate / pre-intervention rate. Interaction term was used for the comparison between primary care physicians and non-primary care physicians. P value for difference is derived from multiple comparison method.
[b] The reference group in the subgroup analysis.
[c] p<0.001 for rate ratio in annual pre-post analysis.
[d] p<0.001 for difference between the group with specified trait and reference group. P value for difference is derived from multiple comparison method.

© 2022 Duan L et al. *JAMA Network Open.*
